# CULTURAL COMPETENCE TRAINING AND EVALUATION AMONG NURSING HOME STAFF

**DOI:** 10.1093/geroni/igae098.3833

**Published:** 2024-12-31

**Authors:** Tochukwu Okolie, Tina Sandri, Staja Booker, Prince Ekoh, Kingsley Udeh

**Affiliations:** Miami University, Oxford, Ohio, United States; Forest HIlls of DC, Washington DC, District of Columbia, United States; University of Florida, University of Florida, Florida, United States; University of Calgary, Calgary, Alberta, Canada; MIAMI UNIVERSITY OHIO, oxford, Ohio, United States

## Abstract

The dynamics of cultural and ethnic identity in healthcare has become more salient in the United States due to the growing population diversity. In particular, significant number of nursing home staff are from diverse sociocultural groups, which necessitates cultural competency in nursing homes in order to provide quality care to older adults. Cultural competency in nursing homes is usually analyzed in terms of the interplay between staff members and the older adults, but this study investigated the relational cultural competency between staff members in the workplace. First, cultural competency training on promoting inclusion and respecting diversity, and communication/teamwork was provided to staff members working in a nursing home in Washington, D.C. To evaluate the level of cultural competency post-training, a semi-structured in-depth interview was conducted with 16 nursing home staff. Participants were purposefully selected to capture variation in cultural identity and ethnicity, national origin, work experience and position at the organization. The interviews were conducted individually and audio-recorded after consent was obtained. The audio files were transcribed verbatim and analyzed with the aid of Nvivo12. Findings show that the training was positively perceived as having impacted their outlook of culture and identity in the workplace. Most of the participants were comfortable with working with individuals from different cultures and identified techniques used in communicating when there are language barriers, including utilizing interpreters and translation tools. Participants recommended that organization’s leadership should create more opportunities for intercultural exchange among staff members through potlucks, and recognizing national holidays of staff members.

